# Modulation of Bacterial Pathogenesis by Oppressive Aging Factors: Insights into Host-Pneumococcal Interaction Strategies

**DOI:** 10.5402/2012/267101

**Published:** 2012-05-17

**Authors:** Pooja Shivshankar

**Affiliations:** Division of Cardiology, Department of Medicine, University of Texas Health Science Center at San Antonio, San Antonio, TX 78229, USA

## Abstract

*Streptococcus pneumonia*, (*Spn*, the pneumococcus), is the leading cause of community-acquired pneumonia (CAP) and is responsible for 15–40% deaths in the elderly worldwide. A primed inflammatory status is a significant risk factor for the increased severity of infectious diseases among the elderly (≥65 years of age). Studies have shown that expression of host receptors that the pneumococci bind to invade the tissues are increased thereby increasing the susceptibility to pneumococcal challenge in aged mice. Cellular senescence, an age-related phenomenon that leads to cell cycle arrest may also contribute to increased inflammation in aged mice. Evidence of cellular senescence in aged lungs of humans and mice adds credits to the concept of inflammaging and enhanced bacterial ligands expression during aging. Furthermore, cell senescence has been shown to occur in age-associated lung pathologies such as idiopathic pulmonary fibrosis (IPF) and chronic obstructive pulmonary disease (COPD) that may predispose the elderly to pathogenic assaults, including *S. pneumoniae*. This review highlights the aspects of: chronic inflammation in the aged population; contribution of cellular senescence to age-associated inflammation and their impact on host receptor expression; and, increased susceptibility of fibrosis and emphysematous lesions-bearing lungs to microbial infections.

## 1. Introduction

**Figure 1:**
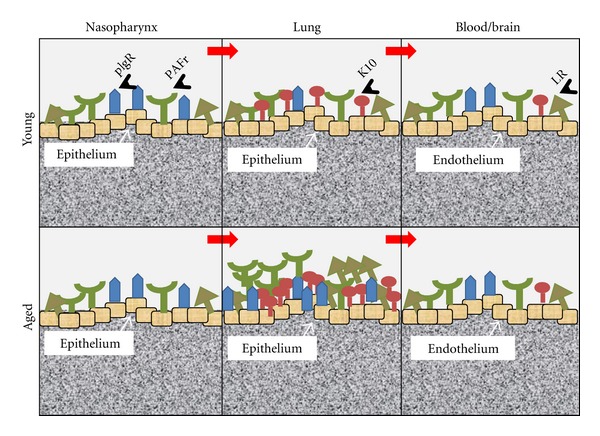
Expression and recruitment of pneumococcal binding proteins (PBPs) on different anatomical sites of the host in the order of pneumococcal binding. Host pneumococcal binding proteins pIgR and PAFr are expressed ubiquitously on epithelial and endothelial cells such as nasopharyngeal mucosal epithelial cells. Pneumococcal adhesin PsrP interacts with K10, besides CbpA and ChoP-mediated binding to pIgR, PAFr, and LR and causes pneumonia. Invasion is facilitated by PAFr, and dissemination occurs resulting in septicemia. Finally, the pneumococcus crosses the blood brain barrier by binging to the PBP, LR on the meningococcal cells, leading to pneumococcal meningitis. Red arrows indicate the sequential binding and invasion of the pneumococcus from the site of colonization to meningococcal invasion. Note the increased expression of PBPs on the lung-cell surface during aging (bottom panel) as compared to the young age.

Aging is a multifactorial process that encompasses progressive decline in multiple organ failure, induced by chronic low-grade inflammation and stress-mediated imbalances. Inflammatory cells such as macrophages, neutrophils, and leukocytes infiltrate various tissues including lungs. Increased systemic levels of proinflammatory mediators such as tumor necrosis factor alpha (TNF*α*) and interleukin-6 (IL-6) increase the risk of microbial assaults in the elderly ≥65 years of age [1, 2]. Community-acquired pneumonia (CAP) is the leading cause of deaths in individuals who are ≥65 years of age [2, 3]. *Streptococcus pneumoniae* is a major cause of CAP among this age group. The annual mortality rate due to CAP among the elderly ranges from 15% to 20%, and the mortality rate might increase as the population of aged individuals would double with respect to the total population, in the next 30 years [4, 5]. Besides cell-wall polysaccharides that mediate attachment to the host cell-surface glycoproteins, pneumococcal virulent proteins function as adhesins during colonization and invasion at multiple host sites such as the nasopharynx, middle ear, the lower respiratory tract, the bloodstream, and, finally, the blood-brain barrier. These adhesins function differentially at different anatomical sites based on the levels of expression and recruitment of host pneumococcal binding proteins (PBPs; Figure 1). We have previously shown that chronic inflammation in aged mice increases expression of PBPs, resulting in increased susceptibility to pneumococcal infection [6]. Age-associated chronic inflammatory diseases such as atherosclerosis [7], diabetes mellitus [8], and arthritis [9] are accounted for the increased pool of proinflammatory mediators. Individuals hospitalized for these comorbidities are at increased risk for development of CAP [1, 2]. Interestingly these chronic inflammatory diseases are reported to have senescent cells in the vicinity of the areas of inflammation [10–12]. While not all autoimmune diseases prevail with age, but diseases such as bullous pemphigoid increases sharply with age and has been associated with cell senescence [13–15].

Cell senescence is an irreversible shutdown of cell division with a concomitant decrease in the rate of apoptosis [16, 17]. As a negative consequence, senescent cells promote malignant transformation by means of the senescence-associated secretory phenotype (SASP). SASP comprises a pool of proinflammatory cytokines, chemokines, proteases, and growth factors [18]. We have recently demonstrated a second negative consequence of SASP as a modulator of NF*κ*B-activated pneumococcal binding protein, platelet activating factor receptor (PAFr), due to the increased levels of IL-6 and IL-8 production in bleomycin-induced senescent type-II pneumocyte cultures [19]. This review presents a comprehensive account of oppressive aging factors such as chronic inflammation, cell senescence and SASP in the aged lungs, and their role in age-associated lung pathologies such as idiopathic pulmonary fibrosis (IPF) and chronic obstructive pulmonary disease (COPD), that are known to increase vulnerability of the aged patients to pneumococcal disease.

## 2. Inflammation Is Associated with Community-Acquired Pneumonia and Invasive Pneumococcal Disease

Yende et al. [2] has reported that individuals aged ≥65 years with increased serum levels of IL-6 and TNF-*α*, are highly susceptible to CAP and IPD, whereas recurrent infection and mortality also depends on these inflammatory markers along with acute-phase protein and C-reactive protein (CRP) [20]. On contrary, circulating IL-6 and IL-10 is assessed as prognostic markers of severity of the disease in the elderly, and these inflammatory indices categorize patients into systemic inflammatory response syndrome (SIRS) and non-SIRS groups [21]. Constitutive NF-*κ*B-mediated transactivation of genes induces expression of proinflammatory cytokines and chemokines in the aged lungs [6, 22, 23]. It has also been shown that aged BALB/cBy mice (19–22 months) exhibited defective toll-like receptors (TLRs) response when these mice were challenged with *S. pneumoniae *[6]. Levels of TNF-*α* and IL-6 in the lung tissues of aged mice were higher as compared to their younger counterparts (4-5 months) and were positively correlated with histologic evidence of chronic inflammation [6, 19]. The inflammatory phenotype of aged mice and susceptibility to pneumococcal infection corroborated with the young cohort instilled with a subchronic dose of TNF-*α* and subsequently challenged with the identical dose of *S. pneumoniae *[6]. 

## 3. Inflammation Increases Pneumococcus-Binding Proteins (PBPs) on Host Cells of the Elderly

We have demonstrated that chronic inflammation in aged mouse lungs stimulates NF-*κ*B-regulated gene expression including the pneumococcus-binding proteins, polymeric immunoglobulin receptor (pIgR), and platelet-activating factor receptor (PAFr). While pIgR binds to the pneumococcal adhesin Choline-binding protein A (CbpA), PAFr binds to phosphorylcholine (ChoP) moiety of the pneumococcal membrane phospholipid [6, 24]. These proteins are found on the upper respiratory tract cells, lung epithelial cells, and endothelial cells of the blood and brain, thereby emphasizing the consequences of pneumococcal pathogenesis in relation to these two very common host receptors. Importantly, pIgR and PAFr are not only opted by the pneumococcus, but they are also receptive to other pathogens such as *Haemophilus influenzae*, *Neisseria meningitidis,* and *Pseudomonas auriginosa* [25–27]. Another receptor known to bind to CbpA is laminin receptor. Laminin receptor (LR) is predominantly present on epithelial and endothelial cells. LR also binds to meningococcal, outer membrane porin (porA) and pilus secretion protein PilQ, and to the *H. influenzae* porin OmpP2 [28]. LR levels were significantly increased in the aged human lung biopsy samples (65–84 years), as compared with their younger counterparts (40–53 years). However, PAFr showed a gradual increase in the protein levels from mature (54–65 years) to aged group human tissue biopsies (65–84 years) versus the young biopsy samples. Aged mice (19–22 months) also displayed significant increase in the levels of PBPs versus their younger counterparts (4-5 months) [19].

A recently discovered pneumococcal adhesin-encoding pathogenicity island *psrPsecY2A2* was correlated with incidence of invasive pneumococcal diseases. The adhesin named pneumococcal serine-rich repeat protein (PsrP) binds to the host microfilament protein and keratin 10 on the lung epithelial cells [29]. K10 is a differentiation marker on keratinocytes, which causes cell-cycle arrest via sequestration of AKT phosphorylation and thence activation of pRb/p107 (homologue of pRb) pathway [30, 31]. Additionally, in chronic, antibiotic-resistant Lyme arthritis, K10, expressed on the endothelial cell layer of synovial blood capillaries, has been shown to act as an autoantigen, and that the autoantibodies generated against K10 lead to chronic arthritis [32]. Therefore, it may be reasoned that K10 not only serves as a ligand for these pathogenic determinants, but also contributes to arresting the cell cycle in alveolar epithelial cells and towards setting up an autoimmunogenic response during vascular tissue damage, resulting in increased inflammation. More importantly, evidence of K10 being expressed on the endothelial cells of blood capillaries indicates a possible involvement of K10 in pneumococcal dissemination into the blood stream, besides LR and PAFr. Given that aged human and mouse lungs express elevated levels of K10, increased attachment of the bacteria to the bronchial and alveolar epithelial cells would be enhanced via K10-PsrP interactions [19, 29]. Preferential binding of the pneumococcus to lung cells of aged mice (19–22 months) remarks K10 as one potential oppressive age-related factor to enhance bacterial pathogenesis and increase susceptibility to pneumococcal pneumonia.

## 4. Cellular Senescence Contributes to Chronic Inflammation and Increased PBPs during Aging

**Figure 2:**
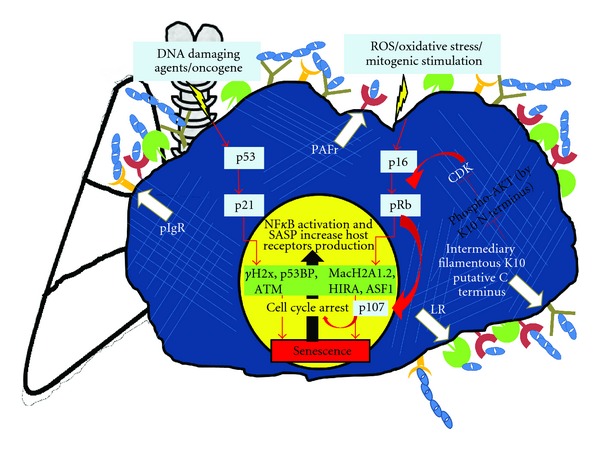
Integral network of cellular senescence and host-pneumococcal interaction in the aged lungs. Lung-cell senescence occurs during the inevitable process of aging. Note the onset of senescence by DNA damage and stress signals is distinctly operated by the two major signaling events, the p53/p21 and the p16/pRb pathways. Both these pathways induce SASP production and enhanced PBPs expression and recruitment. Pneumococcal binding occurs at a comparatively faster pace than under normal conditions, as shown by the binding of the elongated chains of pneumococci. Involvement of K10 as a putative feedback control in mediating cell-cycle arrest is shown as demonstrated by Paramio et al. [30, 31].

Cellular senescence is a dual-edged phenomenon, wherein the cells stop replicating while being metabolically active and do not undergo apoptosis. Senescent cells are inflated with lysosomes with a positive staining for lysosomal *β*-galactosidase (senescence-associated [SA] *β*-gal) activity. The assay was originally performed on old human skin biopsy samples and showed presence of senescent cells on skin fibroblasts and keratinocytes, and till date it is one of the most powerful and authentic assays to confirm senescent phenotypes *in vivo* as well as *in vitro* [33]. Chronic oxidative stress, DNA damage and telomere shortening result in activation of two major tumor suppressor pathways, the p53/p21 and the p16/pRb, pathways that effectively halt gene transcription and promote cell-cycle arrest [34]. Mitogen-activated protein kinase (MAPK) signaling, especially p38-MAPK, is activated independent of DNA damage response in senescent cells, and it is particularly associated with chronic stress-induced inflammation during aging [35]. Activation of these multiple signaling pathways increases NF-*κ*B-regulated transcription of genes including production of senescence-associated secretory phenotype (SASP). According to Coppé et al. [18] SASP is a pool of inflammatory cytokines, chemokines, proteases, matrix metalloproteinases, growth factors, and antiapoptotic factors that help survival of senescent cells and increase tissue consolidation. IL-1*α* serves as the prime regulator of proinflammatory cytokines IL-6 and IL-8 produced during SASP generation [36]. Our studies using A549 type II pneumocyte cultures have shown a second negative consequence of SASP as a promoter of bacterial ligand expression on the normal lung epithelial cells through increased levels of expression of PAFr [19]. We are yet to determine if K10 levels are increased only in native senescent cells and/or transcription factors CEBP*β*/AP-2 that bind to *k10* promoter were not activated effectively in fresh cultures upon the stimulation in SASP for 2 hours [37]. The senescence-inducing proteins network and role of PBPs are illustrated in Figure 2.

## 5. Comorbidities in Aging and Opportunistic Microbial Infection

Comorbidities that run along progressive aging are potential oppressive factors that increase susceptibility of the elderly to pneumococcal infections. For example, elderly with the problems of dental caries and periodontitis become susceptible to oral pathogens that lead to aspiration pneumonia and trigger atherosclerosis [38]. Microbial infections with *Mycoplasma pneumoniae*, and *Chlamydophila pneumoniae* and Cytomegalovirus (CMV) and Epstein-Barr virus (EBV) are encountered in atherosclerotic lesions resulting in exacerbated cardiovascular pathologies [39, 40]. Release of toxic components from *S. pneumoniae* such as pneumolysin, an important pneumococcal toxin, cell-wall polysaccharides, phosphoryl choline (ChoP), and the capsule of the pneumococcus itself, causes severe damage to cardiomyocytes and endothelial cells resulting in plaque formation on the heart valves [41, 42]. Prophylactic treatment with statins has been shown to be beneficial and/or protective against CAP in these patients [43, 44]. Statins are used not only for inflammatory diseases, but also for vascular diseases, due to their immunomodulatory, antioxidative, and anti-coagulation effects [45].

Rheumatoid arthritis (RA) is a chronic inflammatory autoimmune disease with increased risk of opportunistic infections in the elderly. Apart from tuberculosis and leishmaniasis, *Spn*-infected pyogenic muscular abscesses are commonly seen in arthritic patients [46]. A case study from adults with pneumococcal septic arthritis from 1973 to 2003 [47] showed that native joints as well as prosthetic joints were infected with *S. pneumoniae*, which is not surprising because damaged blood vessels might leak the pathogen at various anatomical sites to perpetuate and establish infection. Moreover, treatment with methotrexate (an immunosuppressive agent) in arthritic disease dampens innate immune response and may contribute to increased risk of pneumococcal infection [48].

Chronic inflammation increases the risk of opportunistic infections in diabetes patients. Postinfectious glomerulonephritis is a major concern in elderly patients with diabetes [49]. It was reported that diabetes patients undergoing renal transplantation showed increased incidence of pleural effusion and pneumonia [50]. Nonetheless, diabetes mellitus may not be an independent predisposing factor to pneumonia because generalized oxidative stress and inflammation in diabetic patients also compromise the immune system [51, 52].

Occurrence of cellular senescence has been reported in cardiovascular disease, osteoarthritis, and diabetes mellitus using experimental animal and human tissue biopsies [10–12, 53–56]. Vascular endothelial cell senescence has been demonstrated in atherosclerotic lesions in the rabbit carotid artery [57]. The authors clearly demonstrated vascular endothelial denudation with SA-*β* gal positivity. They further demonstrated mechanisms involved in vascular endothelial cell senescence, such as chronic oxidative stress, telomere shortening, nitric oxide production, and an association between glutathione detoxification system and telomere integrity [57, 58]. Hyperglycemia also increases vascular aging with endothelial cell senescence regulated by apoptosis signal-regulating kinase 1 (ASK-1), supporting the notion that apoptosis inhibition is one of the common ways to accelerate cell senescence [59]. Similarly, chondrocytes were shown to undergo senescence in arthritic lesions of the diseased articular cartilage obtained from aged patients undergoing arthroplasty [60]. Thus, senescent phenotypes contribute to the underlying tissue pathologies and may be implicated in chronic inflammation increased with these age-associated diseases.

## 6. Idiopathic Pulmonary Fibrosis, Cellular Senescence, and Pneumococcal Pneumonia

Idiopathic pulmonary fibrosis (IPF) is a chronic disorder of lungs that affects the elderly. Typically the duration of survival is between 4 and 5 years from the onset of the disease. Worldwide, there are 10.7 cases per 100,000 male populations, and 7.4 cases per 100,000 female populations [61]. IPF patients show poor prognosis due to acute respiratory decline, exacerbated with microbial infections and increasing age, and repeated hospitalization also increases the risk of CAP [62]. Hypothetically, acute exacerbation of IPF has also been associated with reactivation of chronic CMV and EBV infections [63–65]. Interestingly, phenomenon of cellular senescence has been proposed in IPF manifestation [66]. It involves injury to the type II pneumocytes and vascular endothelial cells, and coagulation. At molecular level, greater understanding of the disease has been facilitated, as described by Thannickal and Loyd [67], that epithelial regeneration is curtailed with age and, further relates to telomere shortening, one of the aspects of cell senescence, by Alder et al. [68]. Lymphocytic inflammation of the lungs and foci of proliferating fibroblasts with atypical interstitial pneumonia are used in the diagnosis of pathophysiology of the disease. Biomarkers such as proinflammatory cytokines, chemokines, and MMPs used in the analysis represent senescence indexes.

**Figure 3:**
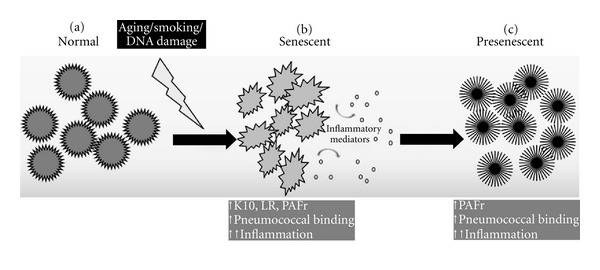
Hypothetic model of cell senescence induction in lung cells due to aging, genotoxic stress, and oxidative stress that causes phenotypic and functional alterations. (a) Normal lung parenchyma is affected by oppressive aging factors, such as DNA damage and oxidative stress, resulting in senescent phenotypes (b) With increased inflammatory mediators production and receptors that facilitate bacterial/bacterial components binding. These mediators affect the neighboring normal cells and alter their phenotypes as presenescent (c). These pre-senescent cells might transform into malignant cell types and induce tumor formation or might eventually become senescent, as proposed by Shivshankar et al. [19].

To understand molecular basis of the disease and study role of cell senescence in lung fibrosis using experimental animals, we administered young Balb/cJ mice (4-5 months) with Bleomycin, a fibrosis-inducing agent [69–71]. Bleomycin-induced lung cell senescence in young mice (4-5 months) showed increased susceptibility of the mice to pneumococcal challenge similar to that of healthy aged mice (19–22 months), along with a significant increase in the levels of p16 and LR levels, with an increasing trend for PAFr. We also demonstrated through an *in vitro* model of senescence induction using bleomycin that senescent cells showed increased levels of PBPs in the order of LR > K10 > PAFr, whereas, as discussed earlier, SASP induced the PBPs in the order of PAFr > LR > K10. Inflammatory cytokines profile was increased in both *in vivo* and *in vitro* studies. Thus, the novel concept of cellular senescence that occurs with progressing age might play a significant role in lung pathologies such as IPF and susceptibility to pneumococcal infection as depicted in the model (Figure 3).

In a recent study by Minagawa et al. [72], *β*-gal-positive senescent epithelial cells and increased levels of p21 were demonstrated in lung biopsies of IPF patients, and also established,* in vitro*, that TGF*β* plays a pivotal role in inducing lung epithelial cell senescence, and that the DNA repair specific sirtuin (SIRT), SIRT6 inhibited TGF*β*-induced senescence. TGF*β* is a pleiotropic growth factor involved in airway remodeling and fibrosis and has been shown to be an integral component of the pathologic network of lung diseases such as asthma and IPF [73, 74]. Supporting Minagawa et al. study, we have recently demonstrated that a membrane-scaffolding protein, caveolin-1, is involved in epithelial cell senescence in mice with bleomycin-induced pulmonary fibrosis [75]. Caveolin-1 has also been implicated in airway remodeling, as an upstream regulatory factor for TGF*β* signaling by sequestering TGF*β* receptor function [76].

It would be worth mentioning that autoimmunity against periplakins has been associated with IPF pathobiology [77, 78]. Bullous pemphigoid (BP), the autoimmune disease caused by periplakins, was directly associated with interstitial pneumonia for the first time, with the presence of IgG and C3 on the basement membranes of lung and skin specimens from a 73-old patient [78]. Interestingly, in children with acute otitis media (AOM), and bullous myringitis (BM), *S. pn *cultures were isolated demonstrating that the pneumococci could be the potential cause of BM, as a severe form of AOM [79]. Given the fact that periplakins interact with keratin filaments, and cell senescence has been implicated in the BP [12–14], it could be speculated that the manifestations of BP on the lung surface may also facilitate pneumococcal binding and invasion of the alveolar mucosal layer and enhance susceptibility to infections in aged patients. Thus, molecular understanding of IPF has been growing in recent years, indicating a possibility of early diagnosis and prevention of the disease.

## 7. Chronic Obstructive Pulmonary Disease, Cellular Senescence, and Pneumococcal Pneumonia

In the elderly, COPD is an important predisposing factor in the incidence of CAP and is currently assessed as a predictor of CAP. Over 90% of the COPD patients worldwide develop CAP, in the age ≥ 65 years, and case fatality rate goes up to 16%–40%. The main risk factor for COPD is smoking [80] although complications such as asthma, environmental stress, and genetic alterations postexposure to the stress factors are debatable in predisposing to COPD [81, 82]. Chronic low-grade inflammation with progressive aging, along with inflammation resulted by COPD, leads to cardiovascular complications worsening the clinical outcome and increased mortality in these patients [83–85]. Mortality rate in CAP patients with COPD is higher by 30–90 days versus the non-COPD patients hospitalized during the same period [86]. Both acute and chronic bacterial infection occurs in patients ≥65 years of age, and the most common infection-causing pathogen is *S. pn *(≤40%), followed by *H. influenzae* and *Chlamydophila pneumoniae * [86, 87]. Sputum samples from COPD patients showed positive cultures for *S. pneumoniae*, *H. influenzae*, and *Moraxella catarrhalis* [88]. Histology readouts include emphysematous lesions in the lungs with loss of airway epithelial mesh and destruction of the walls of the alveoli as some of the manifestations during pathologic examination of the lung biopsies [89, 90]. Interestingly, lung tissue biopsies obtained from COPD patients and animals exposed to smoking showed NF-*κ*B-induced inflammation and also had senescent type II pneumocytes [91, 92]. Along the same line, we have demonstrated in the mouse model that a more generalized oxidative stress in mice induced by hydrogen peroxide-supplemented drinking water promotes cell senescence with epithelial cell injury, alveolar wall destruction, emphysematous lesions, and susceptibility to pneumococcal challenge [19]. Furthermore, we demonstrated that even with severe pathologic destructions of the tissue parenchyma, there was an obvious induction of p16 and pRb expression along with the PBPs LR and PAFr. Pneumococcal burden in the lungs was significantly higher than the control mice and was positively correlated with the senescent phenotype [19], thereby supporting the notion that cellular senescence might be an important contributor of oxidative-stress induced tissue damage during smoking and occupational exposures. 

## 8. Immunosenescence in the Elderly and Defense against Pathogenic Assaults

Immunosenescence is characterized by a decreased production of naïve T and B cells, and increased memory or effecter T and B cells that are differentiated. Studies on the cytomegalovirus (CMV) infection and differentiated memory T-cells function demonstrate the inability of the memory T cells to recognize novel antigens and relates to immunosenescence with age [93]. A defective natural killer cell function and reduced dendritic cell count add to the defective immunity. A very recent review by Kuijpers and Lutter [94] describes how chronic inflammation in a rare congenital disorder, chronic granulomatous disease (CGD) increases the risk of recurrent infections, due to poor phagocytic killing. Thus the whole spectrum of immunosenescence leads to a compromised immune system resulting in increased risk of incidence of infectious diseases such as *S. pn* as well as noninfectious diseases such as dementia, diabetes, and atherosclerosis [95]. While macrophaging is related to inflammaging, studies with toll-like receptor agonist and endotoxins have demonstrated an age-dependent defect in macrophage function in eliciting innate immune response. Consistently, we have also observed that alveolar macrophages isolated from a significantly decreased production of IL-6 in both mature (10–12 months) and aged (19–22 months) mice postinfection with live *S. pn*. Furthermore, results were confirmed *in vitro* with isolated alveolar macrophages stimulated with pneumococcal cell wall fraction and other known TLR agonists (unpublished data). It could be speculated that because IL-6 is required for production of acute phase proteins and to clear the infection by enhancing phagocytic killing, which probably does not occur due to delayed innate immune response and aged mice succumb to infection earlier than the healthy young mice [6]. Given the fact that pneumococcal adhesins PspA and CbpA interfere with the complement system and affect immune adherence and phagocytosis by macrophages [96, 97], it could be reasoned that, in addition to defective alveolar macrophage function, inhibition of complementation and phagocytosis may further affect clearance of the bacteria.

## 9. Conclusions

It is important to understand that the cell-mediated and humoral immune responses function together in a young healthy and immunocompetent system and are considerably impaired with age. In addition to chronic microbial burden in the form of sessile bacterial plaques, other host factors also tend to generate chronic antigenic stress that makes large amounts of memory T cells. Constant generation and expansion of memory T cells, with a decrease in naïve T cells, results in persistent inflammatory status over time and aging [95]. Therefore, per our understanding, if inflammaging is the culprit of the current scenario of elderly individuals being vulnerable to pneumococcal infections with underlying co-morbidities, cellular senescence might be added on to the list of inflammation contributors and as an exceptional oppressive aging factor in the diseased conditions.

Interestingly, pneumococcal adhesins could be proven to be potential candidates in inducing protective immunity and therefore considered in vaccine development. The antibiotic resistant strains of *S. pn* cause most of the serious illnesses in both children and the elderly, and that presence of these adhesins, such as *psrP* in many of these strains particularly, prompts the idea of developing newer/better therapeutics [98]. Recently, protective immunity against one of the most common virulence factor PspA was achieved by parenteral administration of a DNA vaccine [99]. Furthermore, both PspA and CbpA are demonstrated to block phagocytic macrophages adhesion to infected cells by masking the C3 complement system [100]. Considering the presence and virulence potential of PsrP in disease-causing strains, PsrP might be a potential candidate to be included in the conjugate vaccine of the 3rd generation to cover a wide range of carriage-specific or disease-causing *Spn.* The importance of PsrP being a potential vaccine candidate has yet another stand as keratin 10 levels are elevated in aged lungs of humans (55–84 years) and Balb/cBy mice (19–22 months). The only vaccine approved by the Food and Drug Administration (FDA) for protection of adults against pneumococcal diseases is Pneumovax 23 (Merck & Co. Inc). However, Pneumovax 23 does not robustly protect the elderly against pneumococcal pneumonia. Despite prompt vaccinations, elderly patients show vulnerability to infections due in part to an inefficient antibody production and adaptive immune response. Thus, the current scenario warrant a newer 3rd generation conjugate vaccine with virulence proteins as antigenic determinants. Interestingly, vaccination of children ≤ 2 years of age with protein-based polysaccharide vaccine, PCV-7, has resulted in a decline in the incidence of invasive pneumococcal disease among the older adults, due in part to decreased indirect effects of pneumococcal transmission, called herd immunity [101, 102]. The success of children vaccination brought an 18% decrease as compared to the surveillance data from 1995 to 1998 and 1998 to 2001 cohort study [103, 104]. However, additional concerns such as lack of a robust immune system, susceptibility due to hospitalization for comorbid conditions, and influenza infections might endanger patients with secondary infection with *S. pneumonia. *Although IPF patients are advised to receive vaccination against *S. pn* infection to prevent acute exacerbation of IPF, incidence of acute exacerbation of the disease has been reported with H1N1 flu vaccination procedures in these patients [105]. Hence a prompt check has to be enforced in these patients to closely observe the outcomes of vaccination procedures and the severity of the disease pathologies. Anti-inflammatory drugs, along with synthetic telomerase inhibitors, would presumably be a promising choice to protect the elderly with IPF advancement and acute exacerbation by concurrent microbial infections.

Finally, antiaging mechanisms such as the heat-shock proteins, ubiquitination of damaged proteins, ER stress-mediated degradation of proteins, and apoptosis result in clearance of damaged cells and tissue regeneration. These cellular mechanisms can be promoted by activities such as improved dietary schedules with regular exercise, nonsmoking task and a better social life style, which directly impact free radical scavenging, DNA damage repair, balanced energy production and metabolism, and regulated gene expression.

## 10. Future Perspectives

**Figure 4:**
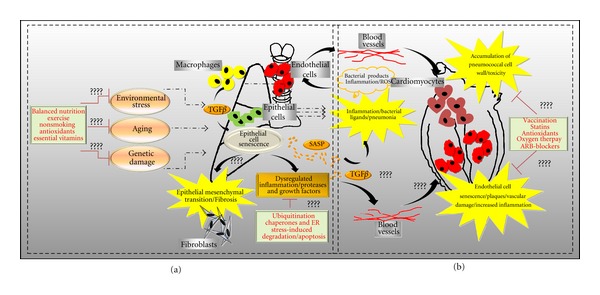
Future perspectives and new lines of research. Representation of perspectives and new lines of research. (a) Demonstration of factors involved in senescence induction in the lungs and susceptibility to pulmonary fibrosis and bacterial infection. (b) Consequences of pneumococcal infection lead to increased inflammation, and toxic bacterial products transmitted through the blood vessels cause cardiomyocyte toxicity. In addition, paracrine effect of SASP cells may increase PAFr production and contribute to endothelial cell senescence in the cardiac tissue, resulting in adverse cardiac events.

Collectively, our previous studies have demonstrated that cellular senescence increases bacterial ligands expression in lung cells and positively correlates with increased pneumococcal binding in aged mice. As discussed above, cell wall component phosphorylcholine (ChoP) released from lysed bacterium, invades vascular endothelial cells, and also damages cardiomyocytes in a PAFr-dependent manner. It is important to understand if paracrine effect of SASP triggers PAFr expression in vascular endothelial cells and cardiomyocytes in cardiac tissue during aging. We therefore hypothesize that endothelial cell senescence may contribute to increased pathology of the cardiac tissue and chronic inflammation resulting in severe cardiovascular events during bacterial infections in the elderly. Finally, TGF*β* is proposed to play a role in inducing alveolar epithelial cell senescence and fibrosis. We will further investigate if TGF*β* is: 1) produced by senescent cells; 2) secreted as a constituent of SASP; and, 3) involved in dysregulated inflammation and triggering endothelial cell senescence in the vasculature during aging (Figure 4).
